# Histopathological Evaluation of the Healing Process of Standardized Skin Burns in Rabbits: Assessment of a Natural Product with Honey and Essential Oils

**DOI:** 10.3390/jcm11216417

**Published:** 2022-10-29

**Authors:** Anis Anis, Ahmed Sharshar, Saber El Hanbally, Awad A. Shehata

**Affiliations:** 1 Department of Pathology, Faculty of Veterinary Medicine, University of Sadat City, Sadat City 32958, Egypt; 2 Department of Surgery, Anesthesiology and Radiology, Faculty of Veterinary Medicine, University of Sadat City, Sadat City 32958, Egypt; 3 Department of Pharmacology, Faculty of Veterinary Medicine, University of Sadat City, Sadat City 32958, Egypt; 4 Avian and Rabbit Diseases Department, Faculty of Veterinary Medicine, University of Sadat City, Sadat City 32958, Egypt; 5 Research and Development Section, PerNaturam GmbH, 56290 Gödenroth, Germany; 6 Prophy-Institute for Applied Prophylaxis, 59159 Bönen, Germany

**Keywords:** regeneration, skin burns, natural, composite, rabbit, histopathology

## Abstract

Skin burns are one of the most difficult medical problems. Recently, studies have been directed towards development of natural products in order to identify effective and safe remedies. In the present study, we evaluated the efficacy of a natural composite (formulated from honey and essential oils) compared with MEBO^®^ (0.25% β-sitosterol) and DERMAZIN^®^ creams (1% silver-sulfadiazine) in the treatment of thermally induced skin burns. For this purpose, four burn-wounds were created on the back of male New Zealand rabbits (*n* = 10) using a thermal stamp under the effect of general anesthesia. Each wound represents one of the following groups: non-treated, natural composite-cream, MEBO^®^-cream, and silver-sulfadiazine treated groups, respectively. Treatments were applied once a day topically until one of these wounds appeared to be healed grossly. The non-treated group received no treatment. Grossly, skin burns have been healed after 28 days of the treatment in all groups except of the non-treated group. The healing efficacy of the natural composite, MEBO^®^ and silver-sulfadiazine creams was quite similar macroscopically. However, microscopically, the epidermal layer of the composite-cream treated group was more mature than those of both MEBO^®^ and silver-sulfadiazine creams treated groups. In conclusion, the tested composite may be a promising effective and inexpensive treatment of skin burns.

## 1. Introduction

Burns are regarded as one of the most serious injuries. Physical impairments or even disabilities, as well as emotional and mental diseases, can result from burns. In developing countries, burns are one of the public health issues [1,2]. The World Health Organization (WHO) reported that each year approximately 11 million people suffer from burn wounds worldwide. About 180,000 of whom die because of such injuries and the non-fatal burn injuries are a leading cause of morbidity [3]. The majority of these cases occur in low- and middle-income countries in African and South-East Asia regions [3]. Additionally, skin burns are one of the complicated affections [4] which recorded in different animals [5,6,7,8]. However, to our knowledge, there is no documented data to measure the number of skin affections in animals, neither locally nor globally. 

Burn injury can be classified according to its severity, depth, and size. Superficial burns (first-degree) are the type of burns that affect the uppermost layer of the skin (epidermis only), and skin becomes red with limited pain. Second-degree burns are divided into two types, superficial partial-thickness burns and deep partial-thickness burns. The superficial partial-thickness burns are characterized by painful, weep, and require dressing and wound care. However, the deep partial-thickness burns are less painful due to partial destruction of the pain receptors and require surgery. The third-degree burns affect the full thickness of the skin and are not typically painful due to the damage to the nerve endings and requires protection from becoming infected. Finally, the fourth-degree burns involve the underlying tissue such as muscle or bone and frequently accompanied by sloughing of burned tissues [9]. 

Skin burns can be induced by thermal, chemical, radioactive and electrical causes. Heat is one of the most common causes of skin burns which can induce a sudden coagulative necrosis of cells and tissues producing physical and physiological damage to the affected tissues resulting in enormous complications if not treated quickly and efficiently. Skin healing and regeneration of the complicated cases of skin burns need long hospitalization, expensive medications, and long rehabilitation programs [10,11,12]. 

Indeed, skin burns are quite common in animals, for example, dermal skin burns in pet animals has been reported after prolonged sun exposure especially in high ambient temperature as plaques and eschars. The microscopic features of theses lesions (like epidermal, vascular, and adnexal necrosis, and subepidermal vesiculation) were consistent with full-thickness thermal skin burns. The brown dachshund dogs are sensitive to direct sunlight exposure, which induce cutaneous skin burn [5,13,14]. Additionally, thermal skin burns have been recorded in small animals, most of these burns are caused by intentional or accidental exposure to a heat source, such as boiling liquids, electric heating pads, flames, hot air dryers, hot metals, and hot lamps [6]. Recently, other causes which induce skin injuries such as transdermal drug delivery has become a growing trend in veterinary medicine. Some substances that help in drug diffusion through the epidermis and subcutaneous tissues are known as penetration enhancers, sorption promoters, or accelerants. However, their uses in veterinary products with a high-absorbed dose may result in adverse side dermatological effects such as toxicological irritations [15].

Antibiotics are widely used in the treatment strategies of skin burns to prevent secondary infection, but unfortunately in some cases antibiotics may induce allergic reactions which delay the healing process [16]. Tissue engineered wound dressings and growth factors are also used in wound healing process but they are relatively too expensive. Therefore, studies for the discovery of new natural compounds that can be used in the healing process of skin burns are still required [10]. Several medicinal plants contain a vast diversity of phytogenic compound with antioxidant, anti-inflammatory and immunomodulatory properties [17] which can be the cornerstone of the future medication of burns.

Several natural products such as honey, rosemary and chamomile oils have many health benefits particularly in skin regeneration process. These natural bioactive substances were previously investigated individually as enhancers of wound healing [18,19,20]. In our previous study, combination between these materials enhanced the wound healing in equines [21]. Honey is one of the oldest known traditional treatments and it was declared in the Holy Quran since 1444 years ago. It has been employed for healing of several skin diseases including ulcers, wounds, eczema, and burns [22]. Honey has anti-inflammatory and antibacterial effects which decreasing edema and exudation. Additionally, honey stimulates tissue regeneration which promotes healing and diminishes the scar size [18]. 

Rosemary (*Rosmarinus officinalis* Linn.) is an aromatic plant which has therapeutic properties and has been used in pharmaceutical and cosmetics industries as well as in the folk medicine. Rosemary has antioxidant and anti-inflammatory properties due to the presence of carnosol/carnosic and ursolic acids. Rosemary has been used in the treatment of inflammatory diseases, wound healing, cancer, skin mycoses, cellulite, alopecia, ultraviolet damage, and aging [19,23,24,25,26,27,28,29]. Chamomile, family *Asteraceae*, is one of the most ancient herbal plants used in the herbal medicinal [30]. It contains several bioactive constituents [31]. Chamomile possesses antioxidant and anti-inflammatory properties in addition to its role in skin protection and regeneration of the necrosed skin [30,31,32,33,34,35,36]. Other oils of plant origin such as sesame oil [22,37,38] and olive oil [38,39] have been subjected to several experimental studies to evaluate their effects on the healing process of skin burns. Sesame oil is considered one of the most important sources of β-sitosterol which is one of the most powerful natural anti-inflammatory [7,40] which used to prevent and treat tissue necrosis [40]. Additionally, olive oil contains several bioactive ingredients which enhance the regeneration of necrosed tissues [8,41,42,43,44,45,46,47]. Recently, a mixture of rosemary and chamomile oils with honey, induced efficient and rapid skin regeneration of equine-skin wounds experimentally and clinically [21]. In this study, we compared the healing efficacy of a natural composite containing rosemary oil, chamomile oil, sesame oil, olive oil and clover flower bee honey to other commercial products on thermally induced skin burns in rabbits.

## 2. Materials and Methods

### 2.1. Commercial Creames and Composite Preparation

Commercial creams used in this study were the MEBO^®^-cream (0.25% β-sitosterol, Julphar, Ras Al-Khaimah, United Arab Emirates) and DERMAZIN^®^-cream (1% silver sulfadiazine, SANDOZ, Cairo, Egypt). The composite (formulated in this study) consists of 80% (*w*/*w*) active ingredients and 20% (*w*/*w*) vaseline base. The active ingredients were a mixture of the following: sesame oil 30%, olive oil 10%, rosemary oil 10% and chamomile oil 10% (Alcaptin Pharm Co., Cairo, Egypt), and clover flower bee honey 20% (Agriculture Ministry, Giza, Egypt).

### 2.2. Composite Preparation

Firstly, honey was stirred on magnetic Stirrer (MGS-1C, Shaoxing, China) at 1500 rpm at 37 °C for 10 min. Then the oil mixtures were added drop by drop until complete mixing. Finally, vaseline (37 °C) was added slowly until complete mixing with the composite. 

### 2.3. Experimental Design

**Figure 1:**
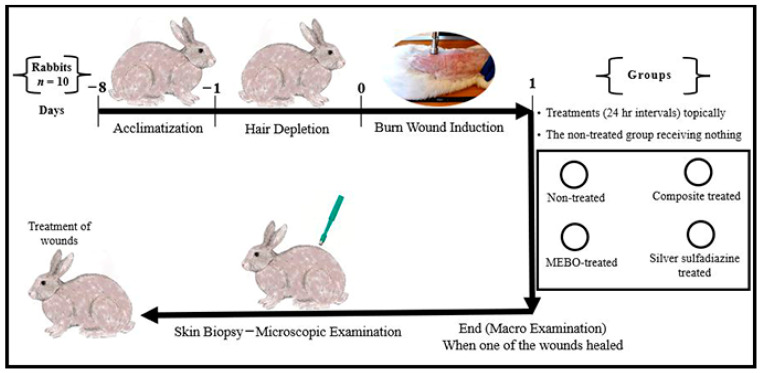
Infograph representing the experimental design. Acclimatization was continued for one week. Hair depletion was done using Veet™ cream topically for three minutes. Burn wound induction was done under the effect of general anaesthesia, four burn-wounds were created by aluminum stamp (80 °C/14 s) on the back of the animal using electronic thermal aluminum stamp represents one of the following treatment groups; non-treated group, natural composite treated group, MEBO^®^ cream treated group, and silver sulfadiazine^®^ cream treated group, respectively. Skin biopsies were collected from the central part of the wounds using disposable skin biopsy punches 10 mm under the effect of general anaesthesia.

Animal-handling procedures as well as sample collection and disposal were done according to the regulations of Institutional Animal Care and Use Committee (IACUC), with approval number: VUSC-004-1-22. It was planned to kill animals humanely if they showed “a generalized dermatitis” or accompanied by complications that cannot be treated. Ten healthy adult male New Zealand Rabbit were used. The mean body weight was 3.6 kg (range from 3.2 to 4 kg). Animals were housed in rabbit cages (one animal per cage) and fed with commercial rabbit diet (Super Rabbit, Egyptian European Company, Cairo, Egypt) while water was dispensed ad libitum along the period of the study. Animal acclimatization was continued for one week, then hair depletion was done using Veet™ (Veet, Cairo, Egypt) cream topically for three minutes. The experimental design is illustrated in Figure 1.

**Figure 2:**
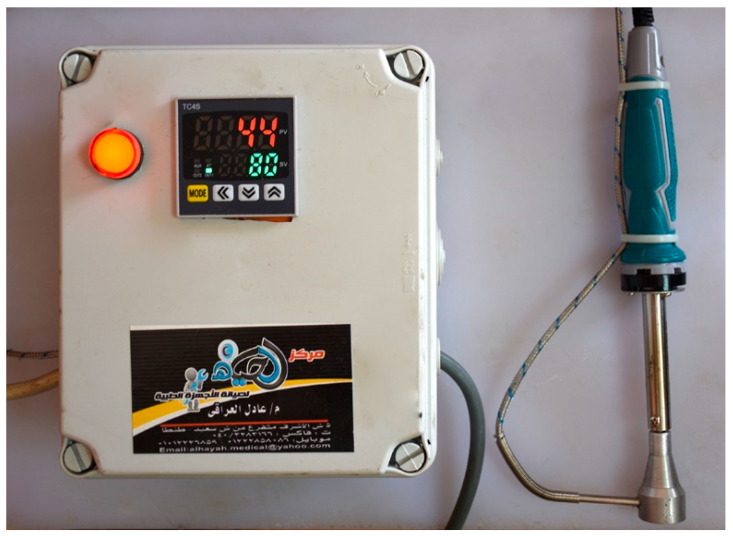
Adjustable electrical aluminum stamp for induction of thermal burn-wounds.

In the next day, anesthesia was induced in rabbits using xylazine and ketamine as method described by Oguntoye et al. [48]. Briefly, rabbits were premedicated first with xylazine (Xylaject^®^: 2% sol. Adwia Company, Cairo, Egypt) at a dose rate of 5 mg/kg. Anesthesia was induced with ketamine (Ketalar^®^: 5% sol. Amoun Company, Egypt) at a dose rate 35 mg/kg. All drugs were injected into *Longissimus dorsi* muscle at different locations. When the animal become anesthetized, four burn-wounds were created by aluminum stamp (80 °C/14 s) on the back of the animal by using electronic thermal aluminum stamp, manufactured locally as described by Knabl et al. [49] with some modifications by addition of electronic thermal controller (Figure 2). 

**Figure 3:**
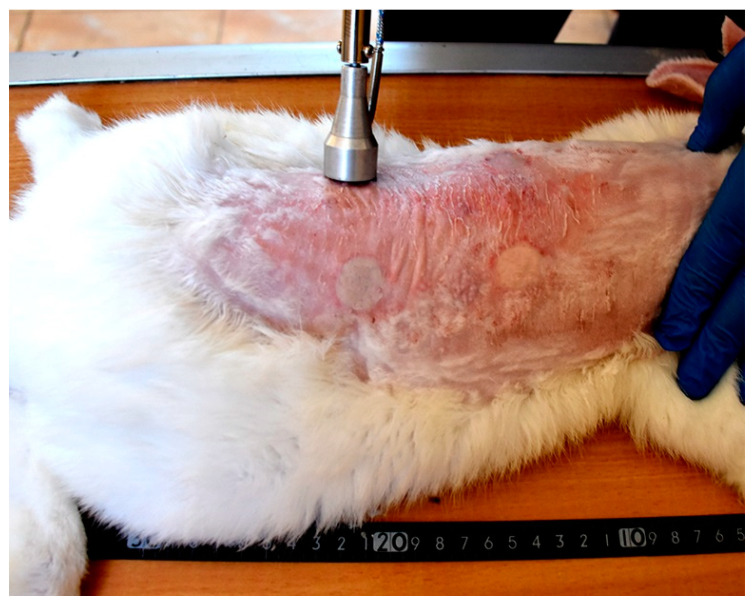
Induction of skin burns in adult New Zealand rabbit. Skin burns were induced using aluminum stamp; the contact area was 4 cm^2^. The site of wounds was anatomically fixed in all rabbits and the distance between the two wounds in the same side was 11 cm, while the distance between wounds from right to left sides was 7 cm.

Each wound from the created four wounds represents one of the following treatment groups; non-treated group, natural composite treated, MEBO^®^ cream treated, and silver sulfadiazine^®^ cream treated groups, respectively. The site of wounds was anatomically fixed in all rabbits and the distance between the two wounds in the same side was 11 cm, while the distance between wounds from right to left sides was 7 cm (Figure 3). 

At the first day of burn induction, skin biopsies were taken from one rabbit at the center of the wound, by using disposable skin biopsy punches 10 mm (MentoK, Jaipur, India), under the effect of general anesthesia for histopathological evaluation of the wounds directly after burn induction. The other nine rabbits were used for the experiment. Treatments were applied once a day topically on the wound and the non-treated group received nothing. The experiment was terminated when one of the wounds appeared to be healed macroscopically. At the end of the experiment, skin biopsies were taken from the center of the wounds, by using disposable skin biopsy punches 10 mm (MentoK, India), under the effect of general anesthesia. After taking skin samples, all rabbits were subjected to intensive medical care to treat wounds created by the biopsy punches. Wounds were sutured and sprayed with betadine until complete healing and the animals were terminated by euthanization.

### 2.4. Histopathological Investigation

Samples were fixed with neutral buffered formalin 10% for 72 h then processed and sectioned at 3–5 um using Leica microtome. Tissues sections were stained with hematoxylin and eosin stain and the histological images were captured using Leica DMLB microscopes and Leica EC3 digital camera.

### 2.5. Morphometery

The morphometric evaluation was done according to methods described previously [50], with some modifications. Briefly, the images were taken with ×20 lens and saved in TIFF format. For each group, nine fields were taken (one field from each animal-skin sample). All fields were taken from the center of the wound. After the fields were saved, the Adobe photoshop program CS6 was used to improve them, using the lighting reference so that they all have the same quality. The full thickness of epidermis and the thickness of stratum corneum were calculated using ImageJ^®^ (Bethesda, MD, USA) program and recorded in a Microsoft Excel spreadsheet.

### 2.6. Statistic Analysis

The data of epidermal measurements are presented as means ± standard error (S.E.) of the means. All morphometric data were subjected to One-way analysis of variance (ANOVA) followed by Duncan’s multiple range test for post hoc analysis using SPSS software, version 17 (IBM, Armonk, NY, USA). The significance level was set at *p* ≤ 0.05. 

## 3. Results

### 3.1. Pathological Evaluation of Skin Burns at the First Day of Induction

**Figure 4:**
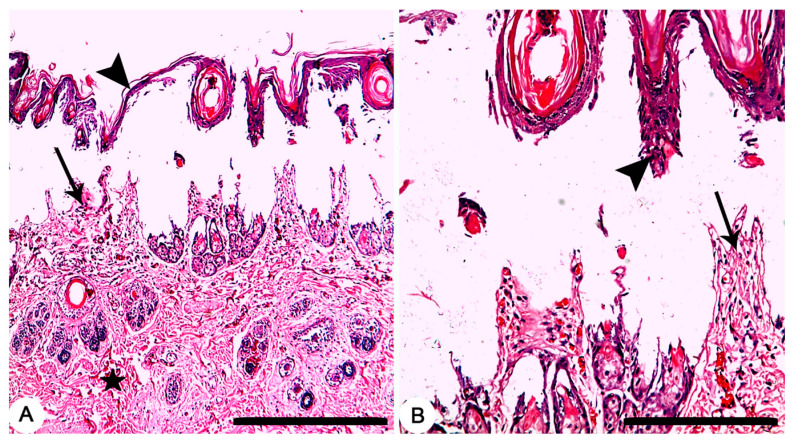
Skin, Rabbit. (**A**,**B**) Thermally induced skin burns using aluminum stamp (80 °C/ 14 s), showing second-degree burns which characterized by necrosis of both epidermal (arrowheads) and dermal (arrows) skin layers with a separation between epidermal and dermal layers, as well as denaturation of dermal collagens bundles (star). HE stain; Bars (**A**) 500 µm; (**B**) 100 µm.

Grossly, circumscribed area of coagulative necrosis which was elevated above the skin surface and take a whitish color (Figure 3). Histopathologically, wounds were recorded as “second-degree burns” (superficial partial-thickness burns). Coagulative necrosis of both epidermal and dermal skin layers was found with a separation between epidermal and dermal layers, as well as denaturation of dermal collagens bundles (Figure 4A,B).

### 3.2. Pathological Evaluation of Skin Burns at the End of the Experiment

**Figure 5:**
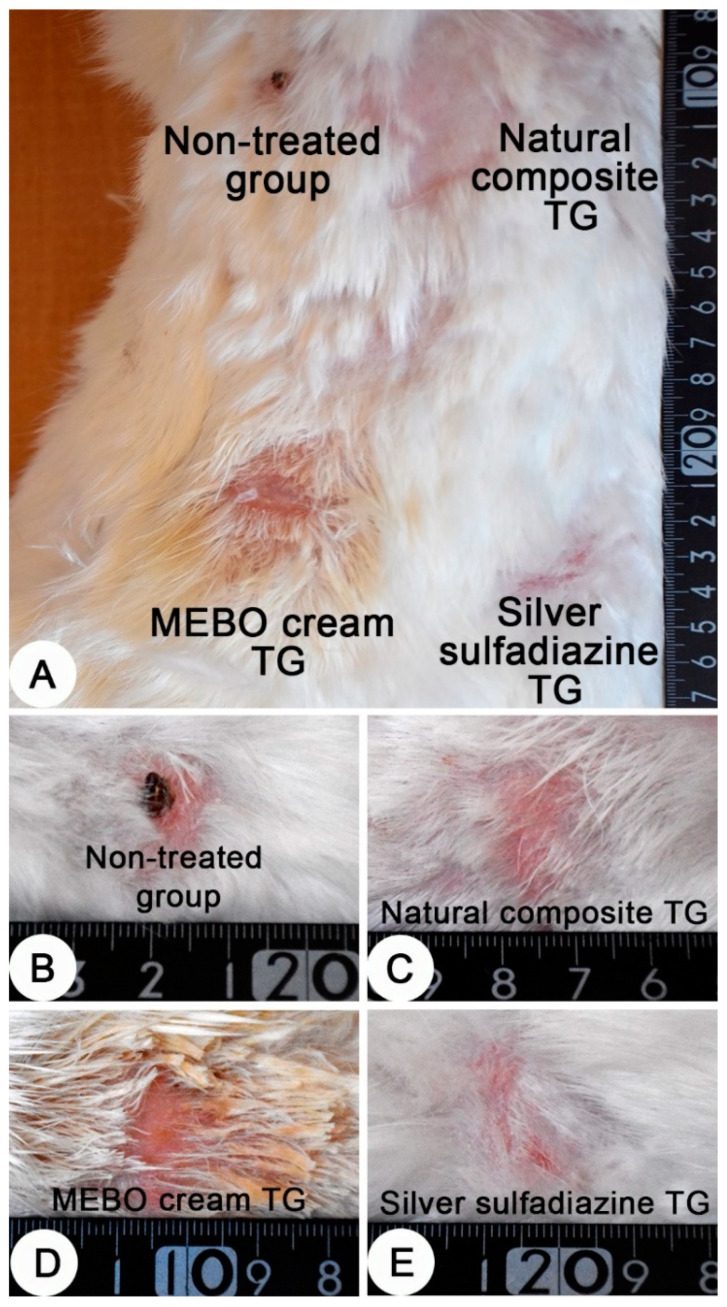
Skin, adult New Zealand rabbit. (**A**) Thermally induced skin burns after 28 days of treatment, showing the four burn sites in one animal. (**B**–**E**) a higher magnification of burn sites of figure A which representing the four treated groups. (**B**) dry crust on the burn site of the non-treated group. (**C**–**E**) complete healing of the burn sites of the treated groups. (**C**) composite cream treated group; (**D**) MEBO^®^ cream treated group and (**E**) silver sulfadiazine cream treated group.

Grossly, after 28 days of the treatment, a dry crust on the burn sites of the non-treated group were observed and a complete healing of the burn sites of the other groups were recorded (Figure 5).

**Figure 6:**
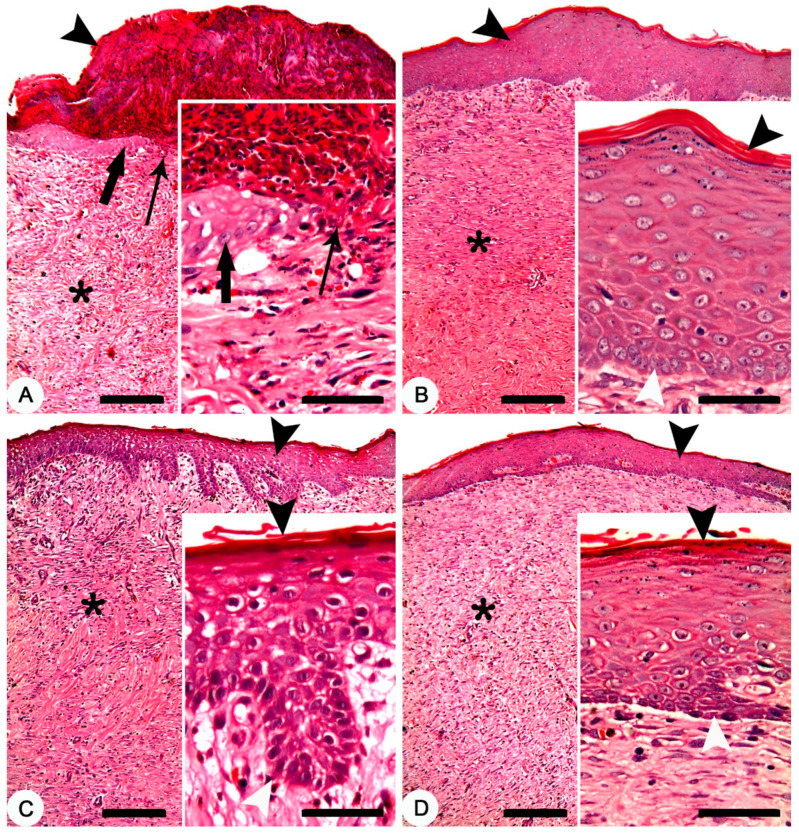
Skin, New Zealand rabbit. Histopathology of thermally induced skin burns after 28 days of treatment. (**A**) non-treated group showing crusts on the surface of the skin (arrowhead), denuded skin surface from the epidermal layer (thin arrow) and a granulation tissue (asterisk). Inset showing crawling of the epidermal cells (thick arrow) over the granulation tissues of the denuded skin (thin arrow). (**B**) composite cream treated group showing complete epidermal layer (arrowhead) covering a granulation tissue (asterisk). Inset showing mature and complete epidermal layers between the stratum corneum (black arrowhead) and stratum germinativum (white arrowhead). (**C**) MEBO^®^ cream treated group showing complete epidermal layer (arrowhead) covering a granulation tissue (asterisk). Inset showing complete epidermal layers between a thin stratum corneum (black arrowhead) and stratum germinativum (white arrowhead). (**D**) silver sulfadiazine cream treated group showing complete epidermal layer (arrowhead) covering a granulation tissue (asterisk). Inset showing complete epidermal layers between a thin stratum corneum (black arrowhead) and stratum germinativum (white arrowhead). HE stain; Bars: main figures 200 µm, Inset 50 µm.

Histopathologically, after 28 days of the treatment, non-treated group exhibited crusts on the surface of the skin. The skin was denuded from the epidermal layer in some parts with advancing epidermal tongue growing over the granulation tissues in other parts (Figure 6A). In composite cream treated group, a mature and complete epidermal skin-layers which covering the granulation tissue was recorded (Figure 6B). In MEBO^®^ cream treated group, a complete epidermal skin-layers, but with a thin stratum corneum, covering the granulation tissue was found (Figure 6C). Finally, a complete epidermal layer, but with a thin stratum corneum, covering a granulation tissue was observed in silver sulfadiazine cream treated group (Figure 6D).

### 3.3. Morphometric Analysis

**Figure 7:**
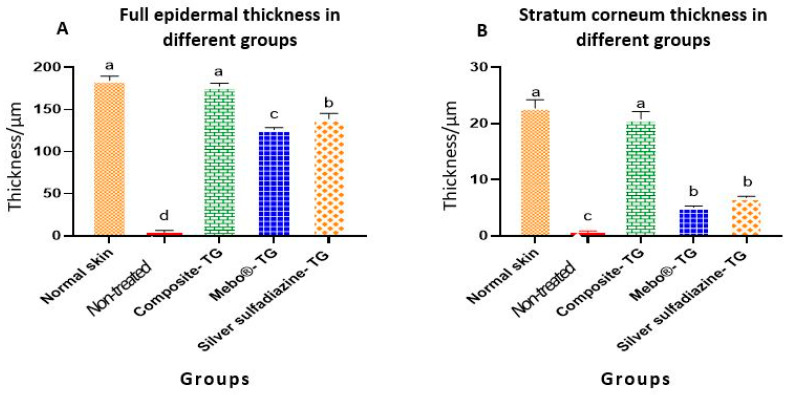
Full epidermal and stratum corneum thickness of normal rabbit skin and different treated groups of experimentally induced skin burns. The measured histological cuts per treatment = 9. Bars with different letters (a, b, c, and d) are significantly different at *p* ˂ 0.05.

Table 1 and Figure 7 present the morphometric changes in the epidermal thickness of normal and different treated groups. According to the data shown, there was no significant differences in epidermal thickness between normal rabbit and composite- treated group. The epidermal thickness of composite-treated group was significantly higher than those of all other groups. Additionally, there was significant different between non-treated group, MEBO^®^-treated group and silver sulfadiazine-treated group (Figure 7A). Finally, the differences of the thickness of stratum corneum of both normal rabbit and composite- treated group was non-significant and both groups were highly significant than all other groups (Figure 7B).

## 4. Discussion

Medication of skin burns are relatively too expensive, especially in poor countries; thus, a discovery of new compounds for skin protection and regeneration are still required [10]. Therefore, we tried to make our efforts to contribute in the discovery of new efficient and cheap phytogenic compounds to accelerate wound healing. This experiment was based on the fact that clover-flower bee honey, rosemary and chamomile oils accelerated wound healing in equines [21]. In this study, we have added sesame oil and olive oil to the above-mentioned composite to make a new composite that can be useful in the treatment of skin burns. Sesame oil is rich in many biological active ingredients especially β-sitosterol [7,8,37,51], which is the active ingredient of a commercially available cream (MEBO^®^-cream). Additionally, olive oil has been used in various previous studies investigating its role in the wound healing process [8,43,52,53].

Herein, the effect of our composite (sesame oil 30%, olive oil 10%, rosemary oil 10% and chamomile oil 10%, clover flower bee honey 20% and Vaseline base 20%) on the skin regeneration process was compared with the most famous commercially available creams used in the treatment of skin burns in Egypt, MEBO^®^ and DERMAZIN^®^. MEBO^®^ is composed mainly of 0.25% β-sitosterol in a base of sesame oil and beeswax [53], while DERMAZIN^®^ is composed of 1% silver sulfadiazine, which was used previously in many studies on the matter of burn healing process [54,55,56,57].

Interestingly, thermally induced skin burns have been healed after 28 days of the treatment except the of negative control group (Figure 5). In all treated groups (except of the non-treated group), the healing process was grossly similar, but histologically, we can distinguish between the different treated groups according to the epidermal structures. In previous studies, MEBO^®^ cream resulted in significant enhancement of wound healing in both healthy and immunocompromised dogs when compared to honey alone and the wound healing effect of MEBO^®^ cream was superior to that of honey [7]. Additionally, silver sulfadiazine was subjected to many studies to evaluate its healing efficacy. It was revealed that silver sulfadiazine accelerated the healing process of burns [56,58]. On the other hand, some plant extracts like *Alpinia officinarum* (galangal) are significantly superior to silver sulfadiazine in the treatment of wound burns [55]. In this study, the healing efficacy of MEBO^®^ cream, silver sulfadiazine cream and our composite were quite similar macroscopically (Figure 5). However, microscopically, the epidermal layer of composite cream treated groups was more mature than those of MEBO^®^ cream and silver sulfadiazine treated groups (Figure 6). In addition, the morphometric analysis (Table 1 and Figure 7) showed that, epidermal thickness of the composite treated group was significantly higher than those of other treated groups. The thickness of stratum corneum of the composite treated wounds in addition to the arrangement and thickness of epidermal layers were quite similar to normal skin structures.

Although the bioactive substances of the natural composite were not analyzed (limitation of this study), the healing efficacy of the composite may be attributed to the nutrients and antibacterial potency of honey [59,60,61], growth promoters and nutrients of both rosemary and chamomile oils [62,63,64], anti-inflammatory and antioxidant properties of sesame oil [7] and vitamins and antioxidants in olive oil. The tested composite characterized by smooth and fast regeneration of the epidermal layers resulting in healthy and histologically uniformed epidermal layers which may be attributed to the several bioactive compounds which present in each component of the tested composite.

## 5. Conclusions

The non-treated group showed a slowed wound healing as compared to the other groups. However, the natural composite promotes the wound healing effectively, highlighting the potential use of natural compounds as effective and safe treatment of skin burns.

## Figures and Tables

**Table 1:** Epidermal thickness of normal rabbit skin and different treated groups (TG) in induced skin burns. Values are presented as mean ± SE (*n* = 9). Different letters (a, b, c, and d) in the same column indicate significant differences at *p* ˂ 0.05.

Groups	Full Epidermal Thickness (µm)	Stratum Corneum Thickness (µm)
Normal skin	184.6 ± 5.09 ^a^	22.8 ± 1.48 ^a^
Non-treated	3.9 ± 2.63 ^d^	0.5 ± 0.35 ^c^
Composite-treated ^1^	177.8 ± 3.39 ^a^	20.8 ± 1.31 ^a^
MEBO^®^ cream-treated ^2^	126.0 ± 2.59 ^c^	5.0 ± 0.27 ^b^
DERMAZIN^®^-treated ^3^	139.0 ± 6.38 ^b^	6.8 ± 0.21 ^b^

^1^ Composite cream: sesame oil 30%, olive oil 10%, rosemary oil 10% and chamomile oil 10% and clover flower bee honey 20%, and 20% (*w*/*w*) vaseline base. ^2^ MEBO^®^-cream = 0.25% β-sitosterol. ^3^ DERMAZIN^®^-cream = 1% silver sulfadiazine.

## Data Availability

Not applicable.
